# The effect of menstrual cycle timing on female elbow flexion force steadiness

**DOI:** 10.14814/phy2.71018

**Published:** 2026-07-12

**Authors:** Cori A. Calkins, Derek C. Chin, Kathryn M. Crosby, Elijah M. K. Haynes, Jennifer M. Jakobi

**Affiliations:** ^1^ School of Health and Exercise Sciences, Healthy Exercise and Aging Laboratory, Faculty of Health and Social Development University of British Columbia Okanagan Kelowna British Columbia Canada; ^2^ Institute of Healthy Living and Chronic Disease Prevention University of British Columbia Okanagan Kelowna British Columbia Canada

**Keywords:** female, force control, forearm position, male, muscle, strength, young

## Abstract

This study examined female elbow flexion force steadiness (*n* = 12) across menses, late follicular, and luteal phases of the menstrual cycle. To control for repeated testing effects unrelated to menstrual phase a male comparison group (*n* = 12) completed the same protocol over three equally spaced sessions as females. Maximal voluntary contractions (MVC) and force steadiness were assessed in neutral and pronated positions. Elbow flexion force tasks were performed at 2.5%, 5%, 10%, 25%, 50%, 75% MVC, and steadiness quantified as the coefficient of variation of force (CV force). Males were stronger than females (*p* < 0.001), and MVC did not differ between the self‐reported menstrual cycle phases (*p* > 0.14) or between days in males (*p* > 0.56). CV of force did not differ across sessions for males (*p* < 0.36). However, in females, CV of force was greater during the luteal phase compared to menses (Neutral *p* = 0.02; Pronated *p* = 0.001); late follicular phase did not differ (Neutral *p* = 0.71; Pronated *p* = 0.10). During the luteal phase, females showed greater CV of force than males in the pronated position (*p* = 0.05). This study identifies reduced upper‐limb force steadiness during the luteal phase (24.5 ± 5.6 menstrual cycle days). This underscores the importance of accounting for menstrual cycle phase when conducting sex‐related comparisons.

## 1 INTRODUCTION

Males typically produce more forceful and steadier voluntary contractions compared to females (Brown et al., 2010; Haynes et al., 2020). These sex‐based differences in force output and modulation could be in part due to menstrual cycle phase whereby cyclical fluctuations of sex hormones occur, which may influence the ability to maintain a steady contraction (force steadiness). Estrogen and progesterone receptors are present in skeletal muscle and throughout the nervous system and are thought to exert neuromodulatory influence on motor unit behavior, which represents the final common pathway to motor output. Estrogen is generally associated with excitatory neuromodulatory effects, whereas progesterone is associated with inhibitory neuromodulatory effects on neural activity (Gargiulo‐Monachelli et al., 2014; Lemoine et al., 2003; Smith et al., 2002). These opposing influences may affect motor unit behavior and neural drive, thereby contributing to menstrual cycle–related fluctuations in force steadiness. The luteal phase is characterized by elevated progesterone levels, which may influence force production and/or increase variability of oscillations in common synaptic input to motor neurons, consistent with its inhibitory neuromodulatory role. Although sex‐based differences in force steadiness have been investigated, far less attention has been given to determining the influence of menstrual phase on force steadiness (Jakobi et al., 2018; Tenan et al., 2016).

A key challenge in this area of research is not the ecological observation of menstrual cycle timing per se, but the assessment and verification of underlying hormonal profiles that fluctuate over the cycle. Direct measurement of ovarian hormones is resource‐intensive and methodologically demanding, requiring repeated sampling, appropriate storage, and costly biochemical analysis (Allen et al., 2016). Stringent exclusion criteria in females for biological factors and/or states such as anovulation can also perpetuate female exclusion in research. There are both perceived and practical challenges associated with hormonal assessment, including laboratory capacity and institutional ethical constraints surrounding repeated blood sampling; the suggested standard for hormonal verification (Elliott‐Sale et al., 2021). These include resource limitations for sample storage and analysis, as well as the high cost of hormone assays, all of which collectively contribute to persistent gaps in female‐specific research (Inglis & Cabral, 2025). Nevertheless, studies that assess force steadiness across the menstrual cycle are valuable. While hormone‐based approaches and day‐specific analyses may provide mechanistic precision, phase‐based testing windows reflect ecologically relevant conditions under which females live, train, and perform. Such approaches capture performance within real‐world temporal contexts rather than strictly laboratory‐defined hormonal states and can provide meaningful insight into time‐related variability in motor performance.

Force steadiness is influenced by various biomechanical factors. For example, position, where a pronated forearm position increases the amplitude of force fluctuations compared to a neutral position (Brown et al., 2010) arising from position‐dependent differences in tendon loading and force transmission within the biceps brachii musculotendinous unit (Smart et al., 2018) and lower motoneuronal excitability in pronation (Yacyshyn et al., 2020). Steadiness also varies with contraction intensity, with the steadiest forces typically occurring at moderate force levels and least steady at low and high force levels (Brown et al., 2010). Accordingly, the contribution of the motor unit pool is also known to vary between low and high forces (Dideriksen et al., 2012; Farina & Negro, 2015), and thus rigorous evaluation of elbow flexion force steadiness across the menstrual cycle should account for both forearm position and contraction intensity.

Despite growing interest in sex‐related differences in force control and modulation (Jakobi et al., 2018; Lulic‐Kuryllo & Inglis, 2022), it remains unknown whether force steadiness varies across the menstrual cycle. This study aimed to determine the influence of menstrual phase on force steadiness in naturally menstruating females not using hormonal contraceptives. A secondary aim was to account for potential effects of repeated testing, ensuring that any observed phase‐related differences were not attributable to repeated testing effects and enabling a sex‐based comparison in force steadiness across self‐reported menstrual cycle phases. It was hypothesized that force steadiness would vary by menstrual phase, with the luteal phase exhibiting the highest CV of force, and that sex‐related differences would be most evident in this phase.

## 2 MATERIALS AND METHODS

### 2.1 Eligibility and recruitment

Young adults between the ages of 19–35 years were recruited to participate in this study. Individuals were excluded if they had a cardio‐metabolic or neurological condition, participated in elite levels of upper‐body resistance training, undergone surgery or sustained a severe injury to the neck, shoulder, or arms, or had taken hormonal contraceptives in the previous 6 months. Female participants were recruited if they self‐reported that they were naturally menstruating and had a consistent cycle length. All participants gave informed written consent before participation. The study was approved by the University of British Columbia Behavioral Research Ethics Board (H16‐00948‐A010).

### 2.2 Experimental set‐up

Participants were seated upright in a custom‐built dynamometer chair that was adjusted such that their hips and knees were flexed at 90°, and their feet were planted on a flat surface. The elbow joint of the dominant arm was flexed at 110° and supported beneath the olecranon. The shoulder was slightly abducted at an approximate angle of 30°. The participant's dominant hand firmly gripped the manipulandum in the pronated (palm down) or neutral (thumb up) forearm positions. A linearly calibrated force transducer (MLP‐150, 68 kg, 266 V sensitivity, Transducer Techniques, Temecula, CA, USA) fixed between the manipulandum and chair arm was used to measure elbow flexion force. The force signal was collected via a bridge amplifier (7.5 V excitation, 266 V sensitivity, Coulbourn Electronics, Allentown, PA, USA) and sampled at 1024 Hz using a Power 1401 analog‐to‐digital converter (Cambridge Electronic Design, Cambridge, England). Elbow flexion force was displayed on a 52 cm monitor 1 m in front of the participants and aligned with a straight line of sight. Force signals were stored locally and analyzed offline (MATLAB, Version 2021A, Massachusetts US).

### 2.3 Experimental protocol

Each participant executed the same protocol over three separate lab visits. Prior to each visit, participants were instructed to refrain from caffeine consumption on the day of testing and to avoid exercise during the 24 h preceding the visit. For females, the three visits occurred based on menstrual cycle phase: menses (Days 1–5), late follicular (Days 6–12), and luteal (day 16 to end of cycle (~Day 28)). The first visit for females was randomized by phase and the subsequent two visits occurred in the natural order of the menstrual cycle following the first visit. The three visits occurred across menstrual cycles depending on which phase they were randomized into on the first visit. Menstrual cycle phase and day were determined from the self‐reported onset of menstruation and were not confirmed using biological measures such as hormone concentrations. Each participant's three visits began within 1 h of the same time of day to mitigate variability due to diurnal menstrual cycle effects (Piirainen et al., 2023). To distinguish if there are any repeated testing session effects on CV of force unrelated to menstrual cycle influence, males also completed three testing sessions over a month. Inter‐visit timing for males was kept as close to females' phase calendar as possible and finalized based on participant availability throughout a month. A separate familiarization session was not included to ensure that the total number of visits and the timing between visits remained consistent with the length of a menstrual cycle. During each visit, participants performed three 5–7 s MVCs in the neutral or pronated forearm position with the initial position randomized between participants. Participants rested for approximately 2 min between each MVC. The highest MVC in each position was used to normalize target force (% MVC). Participants then performed two sets of submaximal tracking tasks at six force levels (2.5%, 5%, 10%, 25%, 50%, and 75%) in a within‐block randomized design. The blocks reflected the neutral and pronated forearm positions, and the block order was also randomized between participants and across days. During these submaximal tracking tasks, participants increased elbow flexion force over a 3‐s ramp to the target force level held for 10 s, and then, over 3 s, lowered force output until full relaxation. The submaximal tracking task was displayed on a monitor and positioned at eye level, and participants were instructed to maintain the target force line. During the experiment, verbal encouragement throughout each task was given.

### 2.4 Data analysis

MVC force was analyzed during each experimental visit (Spike2, version 7.0, Cambridge, UK). The force signals were low pass filtered at 30 Hz and CV of force (standard deviation expressed as a percentage of mean force) during the middle 8 s of the plateau phase was analyzed offline (MATLAB, version 2021A, Massachusetts US). The middle 8 s were chosen to remove any potential overcorrection in force during ramp up or down.

### 2.5 Statistical analysis

To confirm that the number of testing days between visits (1–2, 2–3, and 1–3) did not differ between males and females Welch's *t*‐tests were conducted using software R (R version 4.3.2, The R Foundation for Statistical Computing). The assumption of normality was checked visually with Q‐Q plots. All linear mixed effects (LME) models were conducted using the software R. Prior to each LME model fitting, the extreme outliers were detected with box plots for each dependent variable from the set of observations to be included in the LME model. The values outside the 3rd quartile +3 × interquartile range and 1st quartile—3 × interquartile range were removed from analysis. Q‐Q plots and histograms were used to access the assumptions of linearity, normality and homoscedasticity of the residuals. All residuals were found to be normal and linear.

To assess CV of force for the females between the three phases (menses, follicular, and luteal) at low and high force levels LME models were fit using package lme4 (Bates et al., 2015) with Satterthwaite's method lmerTest (Kuznetsova et al., 2017). Cohen's *d* values between force levels were calculated with effects size (Torchiano, 2020). Phase (i.e., menses, late follicular and luteal) and force level (low (2.5%, 5%, 10%, 25% MVC) and high (50% and 75%)) were coded as fixed effects with participant as a random intercept. Each phase was further compared at each force level (2.5%, 5%, 10%, 25%, 50% and 75%) for each position with separate LME models. Phase was treatment‐contrast coded and force level was sum‐contrast coded. Example model: CV of Force ~ Phase*Force Level + (1|Particpant).

To access CV of force across testing sessions in males, an LME model was fit with day (1, 2, and 3) as a fixed effect and participant as a random intercept. The a priori hypothesis was that males would not differ between days, and subsequently, when statistically proven (see supplemental Figure S1), the first day for the males was used in the subsequent sex‐based LME model to assess CV of force in males (Day 1) to the three phases of the menstrual cycle.

## 3 RESULTS

### 3.1 Phase effects

**FIGURE 1:**
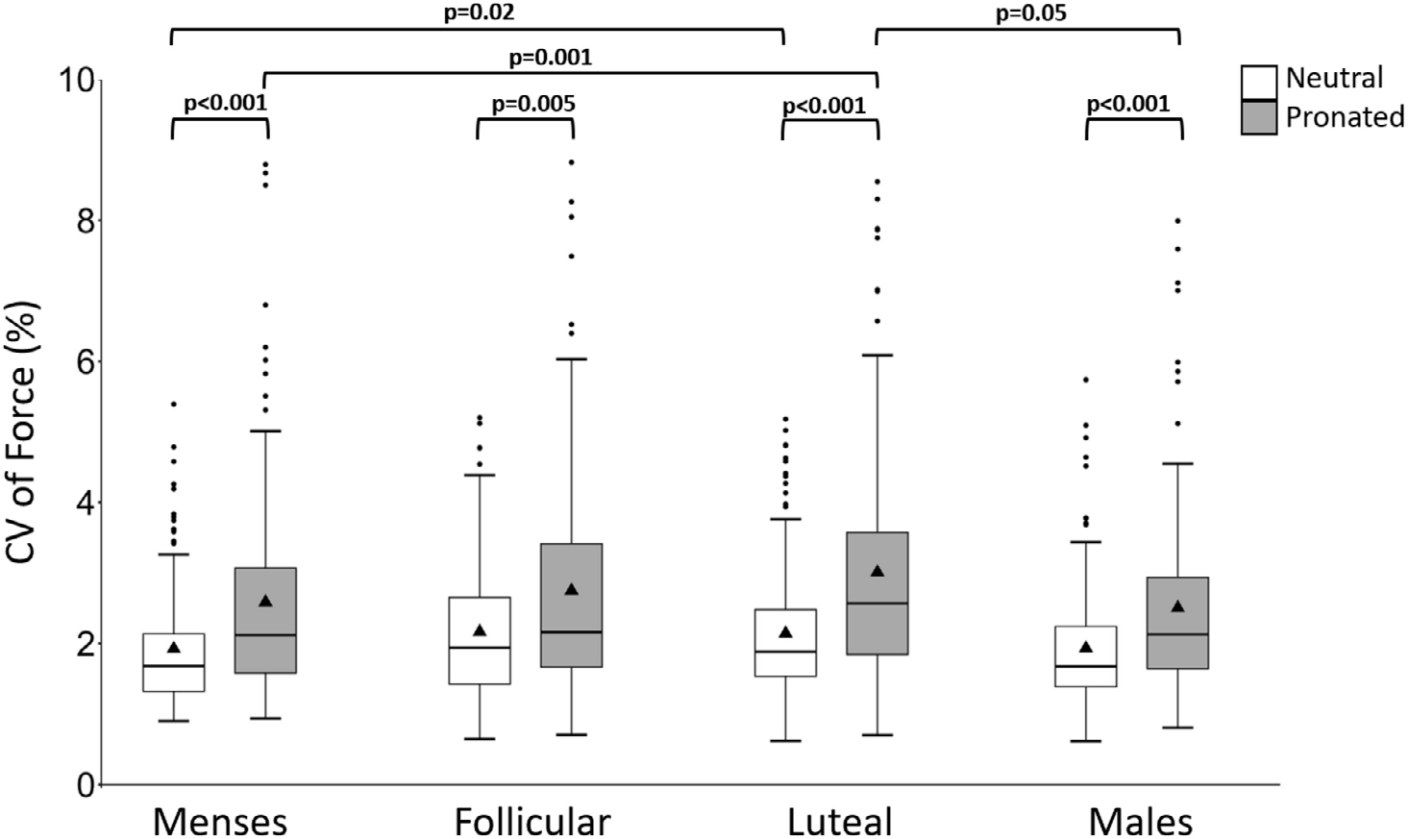
CV of force (%) of females across the menstrual cycle (first three columns) and in the males on their first testing session. No differences were observed in males across the three testing days, which were time matched to the female self‐reported menstrual cycle. The box plots include data from all force levels (i.e. 2.5%, 5%, 10%, 25%, 50% and 75% MVC). The filled triangle denotes the mean. The filled in circles are values above 1.5× the interquartile range.

**FIGURE 2:**
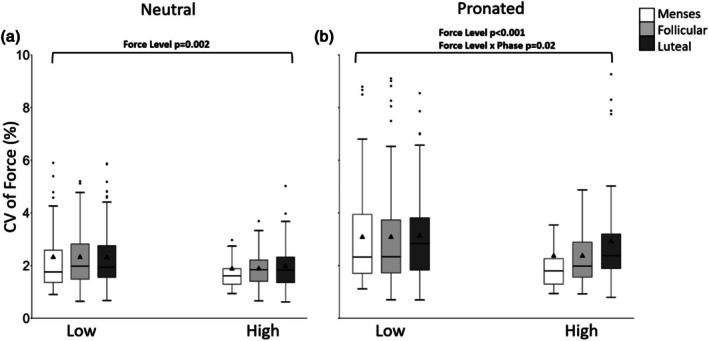
CV of force between low (2.5%, 5%, 10% and 25% MVC) and high (50%, 75% MVC) force levels across the menstrual cycle in the neutral (a) and pronated (b) forearm positions. The filled triangles denote the mean. The phase by force interaction is the difference between menses and the luteal phase between low and high force levels. The filled‐in circles are values above 1.5× the interquartile range.

Twelve females (21.8 ± 2.0 years, 166.8 ± 6.4 cm, 63.8 ± 11.0 kg) and twelve males (22.8 ± 2.2 years, 179.9 ± 5.3 cm, 80.3 ± 14.4 kg) participated in the study. Females visited during menses (3.6 ± 1.4 days), late follicular (9.1 ± 1.9 days) and the luteal phase (24.5 ± 5.6 days). In the neutral (*p* = 0.02) and pronated (*p* = 0.001) position there was an effect of menstrual phase, as CV of force in the luteal phase was greater than menses. There was no significant difference in CV of force between the late follicular phase and menses (Neutral *p* = 0.06; Pronated *p* = 0.11) and late follicular with luteal (Neutral *p* = 0.71; Pronated *p* = 0.10) (Figure 1). There was a significant effect of force level whereby the high forces (50% and 75% MVC) had lower CV of force than the low forces (2.5%, 5%, 10% and 25% MVC) for the neutral (*p* = 0.02) and pronated (*p* < 0.001) position (Figure 2).

**FIGURE 3:**
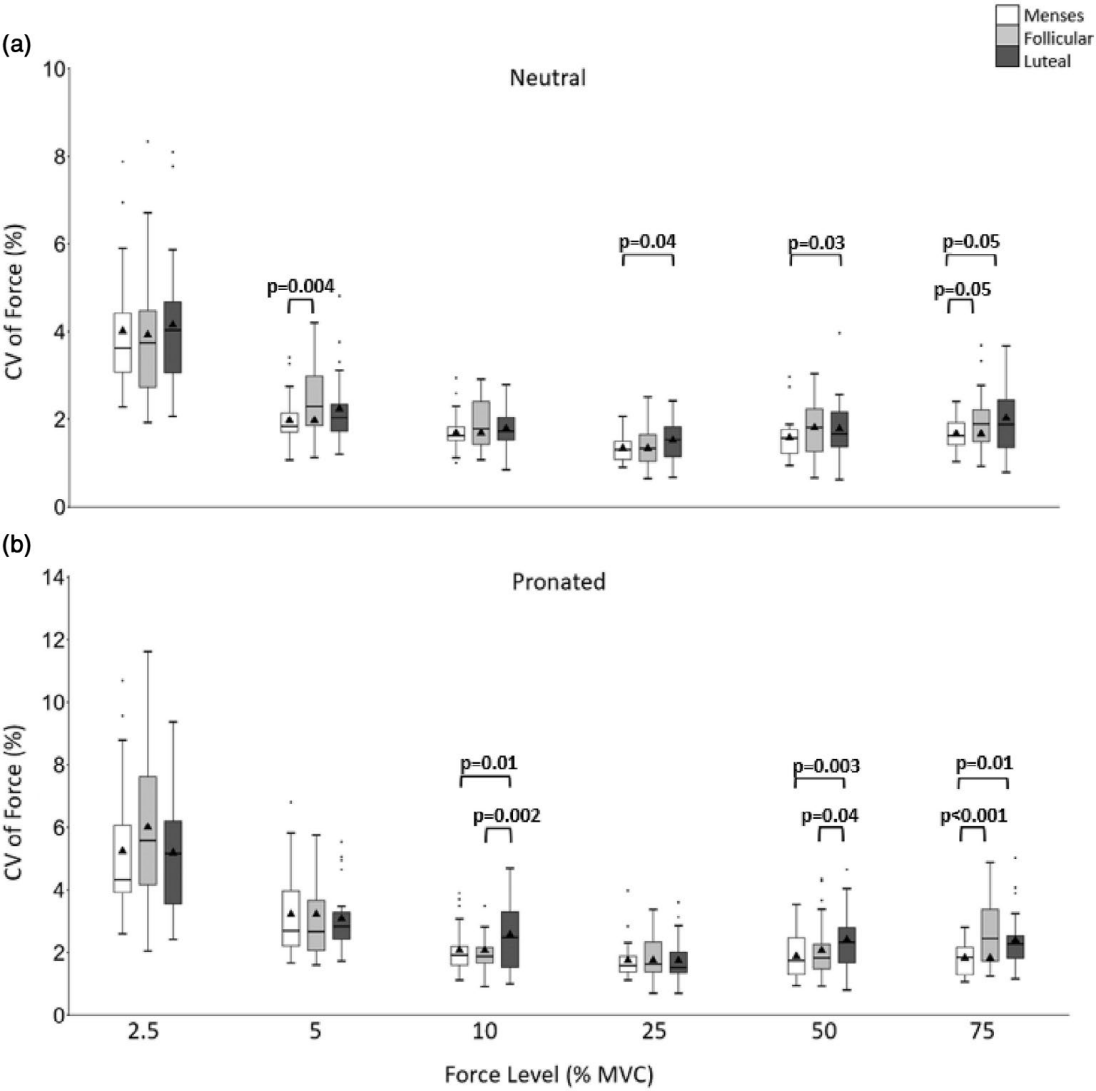
CV of force during isometric elbow flexion at each force level (i.e. 2.5%, 5%, 10%, 25%, 50% and 75% MVC) across the menstrual cycle in the neutral (a) and pronated (b) forearm positions. The filled triangle denotes the mean. The filled in circles are values above 1.5× the interquartile range. The y‐axes differ between (a) and (b) due to pronation being a less steady position. Comparisons are between phases at each force level.

In the neutral position at 5% (*p* = 0.004) and 50% (*p* = 0.05) MVC, the late follicular phase had greater CV of force than menses. In the neutral position at 25% (*p* = 0.04), 50% (*p* = 0.03), and 75% (*p* = 0.05) MVC, the luteal phase had greater CV of force compared to menses (Figure 3a). In the pronated position at 75% (*p* < 0.001) MVC, the late follicular phase had greater CV of force, and at 10% (*p* = 0.01), 50% (*p* = 0.003), and 75% (*p* = 0.01) MVC, the luteal phase had greater CV of force compared to menses. The luteal phase at 10% (*p* = 0.002) and 50% (*p* = 0.04) during the pronated position had greater CV of force compared to the late follicular phase (Figure 3b).

### 3.2 Sex‐related effects

There were no differences found between males and females for the number of testing days between visits 1–2 (males 12.3 ± 6.7 days; females 10.3 ± 5.0 days; *p* = 0.4), 2–3 (males 7.5 ± 5.5 days; females 11.9 ± 7.7; *p* = 0.1) and 1–3 (males 19.8 ± 9.3 days; females 22.2.3 ± 7.5 days; *p* = 0.5). There was no difference in MVC across phase in females (*p* > 0.14) and across day in males (*p* > 0.56) in both forearm positions (Table 1). In neutral and pronated positions, the males had a higher MVC than females (*p* < 0.001). In males, CV of force was lower in the neutral compared to pronated (*p* < 0.001) position. Between days for males in the neutral (*p* < 0.36) and pronated positions (*p* < 0.72) CV of force did not differ. The CV of force in males from day 1 was used to assess sex‐related differences between males and the females in the three self‐reported menstrual phases. The luteal phase in females had significantly higher CV of force in the pronated (*p* = 0.05) position compared to males on Day 1 (Figure 1).

**TABLE 1:** Maximal voluntary contractions across phase or test day in the neutral and pronated positions for females and males.

Testing session	Females MVC (*N*) (*n* = 12)		Males MVC (*N*) (*n* = 12)	
	Neutral	Pronated	Neutral	Pronated
Menses (F); Test Day Me (M)	171.2 ± 33.7*	98.4 ± 29.5^#^	288.2 ± 62.8	188.4 ± 45.6
Late Follicular (F); Test Day Fo (M)	163.7 ± 29.2*	103.7 ± 17.8^#^	283.5 ± 71.5	189.0 ± 56.0
Luteal (F); Test Day L (M)	168.1 ± 33.8*	100.3 ± 23.9^#^	292.4 ± 67.3	190.7 ± 52.4

*Note*: Values ± Standard Deviation. Males were tested at similar intervals of time between sessions and compared to females. Males had a greater MVC in the neutral * and pronated # forearm position. Test day refers to the similar matched time‐point to females.

Abbreviations: F, Female; Fo, Late follicular; L, Luteal; M, Males; Me, Menses.

## 4 DISCUSSION

This study is the first to provide insight into the effects of menstrual phase on force steadiness in naturally menstruating females not using hormonal contraceptives, while also contributing to our understanding of sex‐related differences in force control. The primary hypothesis was supported as force steadiness was poorer during the luteal phase compared to menses in the neutral and pronated forearm positions. Notably, menses was the steadiest and luteal phase showed the poorest force steadiness, particularly at higher force levels. The higher force levels (i.e., 50%, 75% MVC) were steadier than low forces (i.e., 2.5%, 5%, 10%, 25% MVC) and the effects, based upon self‐reported phase, were most evident at greater forces. Furthermore, females in the luteal phase were less steady than males in both the pronated and neutral forearm positions. These findings underscore the importance of accounting for menstrual cycle phase, while evaluating a broad spectrum of contraction intensities and anatomical positions to advance understanding of motor control in females.

Given that MVC is a predictor of force steadiness, it is important to consider maximal strength across the menstrual phase (Brown et al., 2010; Piasecki et al., 2023; Smart et al., 2018). Tenan et al. (2016) observed in the mid‐luteal phase, when progesterone levels peak, knee extension MVC was lowest, and Rodrigues et al. (2019) reported lower strength during menses and luteal compared with the late follicular phase. However, Piasecki et al. (2023) and Michalski et al. (2020) reported no change in MVC across the menstrual phase in the knee and glenohumeral extensors, respectively. Multiple studies, spanning from small to large sample sizes, have employed a range of methodological approaches to understand menstrual cycle effects on MVCs; however, findings remain inconclusive (Wen et al., 2025). This inconsistency may stem, in part, from variability in study design, including differences in phase identification, hormone verification, and testing protocols. Therefore, results should be interpreted with caution. As methodology in menstrual cycle research continues to advance and become more standardized, greater alignment across study paradigms will improve comparability and strengthen interpretation of findings. Although menstrual phase effects on maximal force have been studied, their relevance to force steadiness is not well understood. In this study, elbow flexion MVC did not differ over the menstrual cycle or over testing sessions for males. Therefore, an increase in MVC is not the primary factor contributing to differences in force steadiness across the menstrual cycle or between males and females across the phases.

In females, sex hormone concentrations naturally fluctuate over the menstrual cycle. During menses, estrogen is low and peaks in the late follicular phase, while progesterone remains low throughout the follicular phase (Allen et al., 2016). During the luteal phase, progesterone peaks and estrogen has a secondary lower peak relative to the follicular phase (Allen et al., 2016). Estrogen exerts an excitatory effect on the nervous system, whereas progesterone produces an inhibitory influence (Smith et al., 2002). Overall, these results suggest that force control is optimized during menses, when both hormone levels are low, or in the late follicular phase, which occurs when estrogen is elevated without a concurrent rise in progesterone. Thus, progesterone could be contributing to neuronal inhibition or suppressed excitation; during the luteal phase in animal models, progesterone inhibits motor neuron excitability (Callachan et al., 1987). Inhibition, due to cyclical hormone change, could culminate in a reduction of strength and/or an increase in the variability of oscillations in common synaptic input to motor units, which is associated with reduced force steadiness (Pereira et al., 2019). Force steadiness at high forces, but not low forces, is also most affected by common low frequency oscillations in motoneuron discharge rates (Dideriksen et al., 2012) and potentially contributes to the CV of force being lower in menses than the late follicular and luteal phases at the highest forces of 50% and 75% MVC. To better understand whether hormonal fluctuations across the menstrual cycle directly influence force steadiness, future research should investigate various force levels, anatomical position, and common input to the motoneuron pool in relation to various hormone concentrations. As hormone assessment techniques become more accessible, they may help reduce existing knowledge gaps; however, ecologically valid studies based on self‐reported menstrual cycle timing will continue to provide important insight into neuromuscular performance in females.

For both females and males, the CV of force was greatest at the lowest force levels, gradually declined through moderate force output and then showed a slight increase at the highest force levels (see supporting Cohen's *d* Tables S1–S8 for changes across force). The inverted J‐shaped pattern in force steadiness across force levels was evident at each phase, and strength of comparisons was demonstrable with a large effect size (Cohen's *d* > 2) between the lowest and highest force levels. This substantial effect indicates that regardless of self‐reported menstrual phase, force steadiness is markedly influenced by force level, with moderate force being the steadiest. However, during the high force levels, force steadiness was greater during menses (CV of force lower) than during the luteal phase. Functionally, these findings may have implications for sports that require high force production combined with precise force control to maximize performance. Also, as females age, the potential hormonal influence observed during the luteal phase may diminish following menopause.

The pattern of force steadiness observed in males between the two forearm positions is consistent with previous findings of isometric elbow flexion. As expected, force steadiness was less in the pronated position compared to the neutral position (Kohn et al., 2018; Smart et al., 2018; Yacyshyn et al., 2020). Importantly, force steadiness in males did not change across testing days, suggesting that the ability to maintain a steady contraction was not influenced by practice effects or repeated exposure. Therefore, the differences in force steadiness observed in females across the menstrual cycle or between sexes are unlikely to be attributed to repeat testing.

The higher CV of force in females compared to males during the luteal phase might be due to the progesterone levels. Previously, when progesterone was measured (21.4 ± 5.4 ng/dL) 10 to 3 days prior to the luteinizing hormone peak the progesterone levels did not differ from males (18.1 ± 3.1 ng/dL) (Zumoff, Miller, Levin, et al., 1990). Levels of progesterone rise substantially in the luteal phase; reaching approximately 1500 ng/dL (Zumoff, Miller, Levit, et al., 1990). This may contribute to reduced force control in females. Therefore, a similar level of neuronal inhibition may occur in males and in females during menses and the follicular phase, when female progesterone concentrations are relatively low. Overall, these results indicate that sex‐related differences in force control when participants self‐report menstrual cycle is likely attributed to elevated progesterone levels specifically in the luteal phase.

A limitation of this study is that ovulation was not detected to specifically identify phases, nor were sex hormone concentrations quantified. A study comparing multiple methods for determination of anovulation using serum or urinary analysis reported detection rates ranging from 3.4–18.6% depending on the method used (Lynch et al., 2014), and Malcolm and Cumming (2003) have suggested that definitive confirmation of ovulation may only be possible through ultrasonography. Thus, long phase windows were used to increase the likelihood of capturing the intended phases, late follicular and luteal. The influence of specific hormone concentrations warrants further investigation. Individual estrogen and progesterone levels can vary within and across menstrual cycles. Hackney et al. (2025) suggest that this variability could influence the reproducibility of results and make it difficult to definitively apply findings to the real world. It is unknown whether phase‐related elevations, compared with peak hormone levels, produce similar or attenuated effects on force control. Future studies should consider measuring hormone concentrations at multiple time points within phases and across cycles, as females do not function solely on specific days that correspond to peak hormone levels. Alternatively, researchers may improve real‐world application by exploring overall menstrual phase trends alongside hormone levels.

This study provides novel evidence that self‐reported menstrual cycle phase influences force steadiness. The luteal phase, characterized by elevated progesterone, is least steady and during this phase the effects of force steadiness are particularly evident at higher contraction intensities. These findings highlight the value in considering menstrual phase, and when possible, hormonal levels when evaluating sex‐related differences. While males demonstrated consistent force steadiness across sessions, the variability observed in females across phases suggests a hormonally mediated modulation of motor unit behavior, potentially through changes in common inputs to motor units. Importantly, this work reinforces the value of studying females across the menstrual cycle with clear reporting of testing phase, and highlights the importance of within‐phase comparisons in sex‐based analyses, supporting the integration of both sex‐specific and phase‐specific considerations in neuromuscular research. Given this, it is imperative that future studies should directly assess hormone concentrations and neural mechanisms such as synaptic input variability to better understand how cyclical hormonal changes influence motor control across force outputs and life stages. Overall, these results have implications for explaining menstrual phase trends in force control which occur over daily living in naturally menstruating females.

## AUTHOR CONTRIBUTIONS

**Cori A. Calkins:** Formal analysis; methodology. **Derek C. Chin:** Data curation; project administration. **Kathryn M. Crosby:** Conceptualization; formal analysis; methodology. **Elijah M. K. Haynes:** Data curation; formal analysis; methodology; project administration; supervision. **Jennifer M. Jakobi:** Conceptualization; data curation; funding acquisition; methodology; project administration; resources; supervision.

## FUNDING INFORMATION

Research Support from the Natural Sciences and Engineering Research Council Discovery Grant Program (Grant No: 312038).

## CONFLICTS OF INTEREST STATEMENT

Authors disclose there are no conflicts of interest.

## ETHICS STATEMENT

This study is in accordance with the Declaration of Helsinki, although it was not registered. All participants gave informed written consent before participation. The study was approved by the University of British Columbia Behavioral Research Ethics Board (H16‐00948‐A010).

## Supporting information


**Table S1.** Cohen's *d* values between force levels during menses in the pronated position.
**Table S2.** Cohen's *d* values between force levels during the follicular phase in the pronated position.
**Table S3.** Cohen's *d* values between force levels during the luteal phase in the pronated position.
**Table S4.** Cohen's *d* values between force levels during menses in the neutral position.
**Table S5.** Cohen's *d* values between force levels during the follicular phase in the neutral position.
**Table S6.** Cohen's *d* values between force levels during the luteal phase in the neutral position.
**Table S7.** Males Cohen's *d* values between force levels in the neutral position.
**Table S8.** Males Cohen's *d* values between force levels in the pronated position.

**Figure S1.** Males coefficient of variation of force during elbow flexion across three testing days in the neutral and pronated forearm positions. The bar plots include data from all force levels (i.e. 2.5%, 5%, 10%, 25%, 50% and 75% MVC).

## Data Availability

Data will be available upon request.
