# Supplementary material for: The effect of menstrual cycle timing on female elbow flexion force steadiness
Source: Physiol Rep. 2026 Jul 12;14(13):e71018. doi: 10.14814/phy2.71018 (PMC13358017; doi:10.14814/phy2.71018)
Supplement: Supplementary file 1 — Table S1. Cohen's d values between force levels during menses in the pronated position. Table S2. Cohen's d values between force levels during the follicular phase in the pronated position. Table S3. Cohen's d values between force levels during the luteal phase in the pronated position. Table S4. Cohen's d values between force levels during menses in the neutral position. Table S5. Cohen's d values between force levels during the follicular phase in the neutral position. Table S6. Cohen's d values between force levels during the luteal phase in the neutral position. Table S7. Males Cohen's d values between force levels in the neutral position. Table S8. Males Cohen's d values between force levels in the pronated position. [file PHY2-14-e71018-s002.pdf]

Supplementary Material

Supplemental Table 1. Cohen's d values between force levels during menses in the pronated position

Force Level	5% MVC	10% MVC	25% MVC	50% MVC	75% MVC
2.5% MVC	d=1.1<br>CI=0.43-1.67	d=1.9<br>CI=1.2-2.5	d=2.1<br>CI=1.3-2.8	d=2.0<br>CI=1.3-2.7	d=2.0<br>CI=1.3-2.8
5% MVC		d=1.0<br>CI=0.4-1.6	d=1.3<br>CI=0.7-2.0	d=1.2<br>CI=0.6-1.8	d=1.3<br>CI=0.7-1.9
10% MVC			d= 0.5<br>CI=(-0.1)-1.1	d=0.3<br>CI=(-0.3)-0.9	d=0.4<br>CI=(-0.2)-1.0
25% MVC				d=(-0.2)<br>CI=(-0.8)-0.4	d=(-0.1)<br>CI=(-0.7)-0.5
50% MVC					d=0.1<br>CI=(-0.5)-0.7

Supplemental Table 2. Cohen's d values between force levels during the follicular phase in the pronated position

Force Level	5% MVC	10% MVC	25% MVC	50% MVC	75% MVC
2.5% MVC	d= 1.6<br>CI=0.9-2.3	d=2.3<br>CI=1.6-3.1	d=2.4<br>CI=1.6-3.1	d=2.2<br>CI=1.4-2.9	d=1.8<br>CI=1.1-2.5
5% MVC		d=1.1<br>CI=0.5-1.7	d=1.2<br>CI=0.5-1.8	d=0.8<br>CI=0.2-1.4	d=0.3<br>CI=(-0.3)-0.8
10% MVC			d=0.2<br>CI=(-0.4)-0.8	d=(-0.1)<br>CI=(-0.7)-0.4	d=(-0.8)<br>CI=(-1.4)-(-0.2)
25% MVC				d=(-0.3)<br>CI=(-0.8)-0.3	d=(-0.9)<br>CI=(-1.5)-(-0.3)
50% MVC					d=(-0.6)<br>CI=(-1.2)-0.0

Supplemental Table 3. Cohen's d values between force levels during the luteal phase in the pronated position

Force Level	5% MVC	10% MVC	25% MVC	50% MVC	75% MVC
2.5% MVC	d= 1.4 CI=0.7-2.0	d=1.7 CI=1.0-2.4	d=2.4 CI=1.6-3.2	d=1.8 CI=1.1-2.6	d=1.8 CI=1.1-2.5
5% MVC		d=0.5 CI=(-0.1)-1.1	d=1.5 CI=0.8-2.2	d=0.7 CI=0.1-1.3	d=0.7 CI=0.0-1.3
10% MVC			d=0.9 CI=0.2-1.5	d=0.2 CI=(-0.4)-0.8	d=0.2 CI=(-0.4)-0.8
25% MVC				d=(-0.8) CI=(-1.4)-(-0.2)	d=(-0.8) CI=(-1.4)-(-0.1)
50% MVC					d=0.0 CI=(-0.6)-0.6

Supplemental Table 4. Cohen's d values between force levels during menses in the neutral position

Force Level	5% MVC	10% MVC	25% MVC	50% MVC	75% MVC
2.5% MVC	d= 1.9 CI=1.2-2.6	d=2.2 CI=1.5-3.0	d=2.7 CI=1.9-3.5	d=2.3 CI=1.6-3.1	d=2.3 CI=1.6-3.1
5% MVC		d=0.5 CI=(-0.1)-1.2	d=1.3 CI=0.7-2.0	d=0.7 CI=0.1-1.3	d=0.6 CI=0.0-1.2
10% MVC			d=0.9 CI=0.3-1.5	d=0.2 CI=(-0.4)-0.8	d=0.1 CI=(-0.5)-0.6
25% MVC				d=(-0.6) CI=(-1.2)-0.01	d=(-0.9) CI=(-1.6)-(-0.3)
50% MVC					d=(-0.2) CI=(-0.8)-0.4

Supplemental Table 5. Cohen's d values between force levels during the follicular phase in the neutral position

Force Level	5% MVC	10% MVC	25% MVC	50% MVC	75% MVC
2.5% MVC	d= 1.2 CI=0.6-1.8	d=1.8 CI=1.1-2.4	d=2.2 CI=1.5-3.0	d=1.8 CI=1.1-2.5	d=1.7 CI=1.0-2.4
5% MVC		d=0.8 CI=0.2-1.4	d=1.6 CI=0.9-2.3	d=0.9 CI=0.2-1.5	d=0.7 CI=0.1-1.3
10% MVC			d=0.9 CI=0.3-1.6	d=0.1 CI=(-0.5)-0.7	d=(-0.1) CI=(-0.7)-0.5
25% MVC				d=(-0.8) CI=(-1.4)-(-0.1)	d=(-1.0) CI=(-1.6)-(-0.4)
50% MVC					d=(-0.2) CI=(-0.8)-0.4

Supplemental Table 6. Cohen's d values between force levels during the luteal phase in the neutral position

Force Level	5% MVC	10% MVC	25% MVC	50% MVC	75% MVC
2.5% MVC	d= 1.5 CI=0.8-2.2	d=2.0 CI=1.3-2.8	d=2.3 CI=1.5-3.0	d=1.9 CI=1.2-2.7	d=1.7 CI=1.0-2.3
5% MVC		d=0.6 CI=0.1-1.2	d=1.0 CI=0.4-1.7	d=0.6 CI=(-0.02)-1.2	d=0.2 CI=(-0.4)-0.8
10% MVC			d=0.5 CI=(-0.1)-1.1	d=0.01 CI=(-0.6)-0.6	d=(-0.3) CI=(-0.9)-0.3
25% MVC				d=(-0.4) CI=(-1.0)-0.2	d=(-0.7) CI=(-1.3)-(-0.1)
50% MVC					d=(-0.3) CI=(-0.9)-0.3

Supplemental Table 7. Males Cohen's d values between force levels in the neutral position

Force Level	5% MVC	10% MVC	25% MVC	50% MVC	75% MVC
2.5% MVC	d= 1.9 CI=1.2-2.6	d=2.1 CI=1.3-2.8	d=2.5 CI=1.7-3.3	d=1.7 CI=1.0-2.4	d=1.7 CI=1.0-2.4
5% MVC		d=0.3 CI=(-0.3)-1.9	d=1.1 CI=0.5-1.7	d=(-0.4) CI=(-1.0)-0.2	d=(-0.2) CI=(-0.8)-0.4
10% MVC			d=0.8 CI=0.2-1.4	d=(-0.7) CI=(-1.3)-(-0.1)	d=(-0.5) CI=(-1.1)-0.1
25% MVC				d=(-1.5) CI=(-2.2)-(-0.9)	d=(-1.2) CI=(-1.8)-(-0.5)
50% MVC					d=0.1 CI=(-0.4)-0.7

Supplemental Table 8. Males Cohen's d values between force levels in the pronated position

Force Level	5% MVC	10% MVC	25% MVC	50% MVC	75% MVC
2.5% MVC	d= 1.1 CI=0.4-1.7	d=1.6 CI=1.0-2.3	d-2.0 CI=1.3-2.7	d=1.7 CI=1.0-2.4	d=1.6 CI=0.9-2.3
5% MVC		d=0.8 CI=0.2-1.4	d=1.3 CI=0.7-2.0	d=0.9 CI=0.3-1.5	d=0.8 CI=0.2-1.4
10% MVC			d=0.7 CI=0.1-1.3	d=0.1 CI=(-0.4)-0.7	d=0.1 CI=(-0.5)-0.7
25% MVC				d=(-0.6) CI=(-1.3)-0.0	d=(-0.6) CI=(-1.2)-0.0
50% MVC					d=(-0.1) CI=(-0.7)-0.5
