# Supplementary material for: The effect of menstrual cycle timing on female elbow flexion force steadiness
Source: Physiol Rep. 2026 Jul 12;14(13):e71018. doi: 10.14814/phy2.71018 (PMC13358017; doi:10.14814/phy2.71018)
Supplement: Supplementary file 2 — Figure S1. Males coefficient of variation of force during elbow flexion across three testing days in the neutral and pronated forearm positions. The bar plots include data from all force levels (i.e. 2.5%, 5%, 10%, 25%, 50% and 75% MVC). [file PHY2-14-e71018-s001.docx]

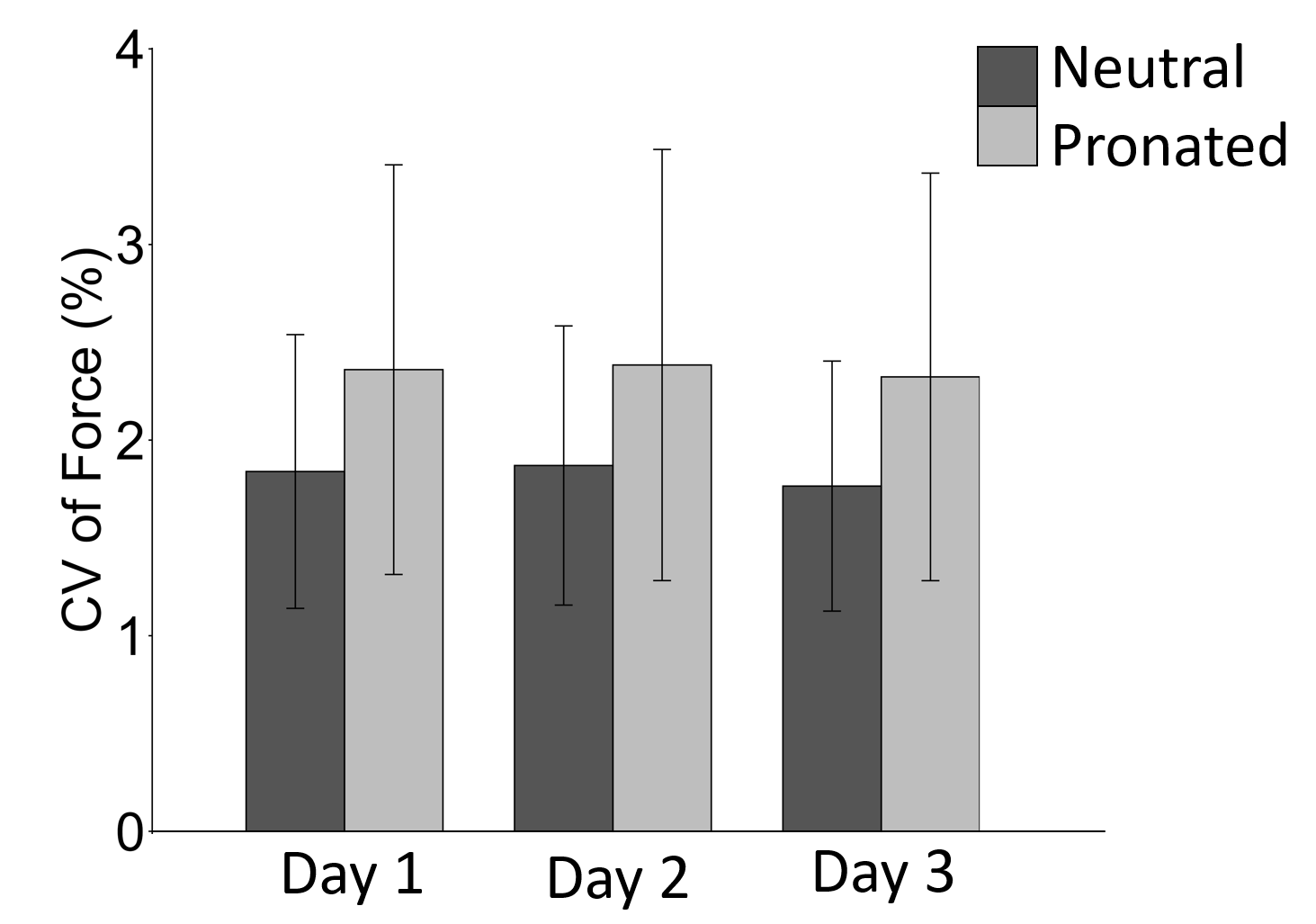


Supplemental Figure 1. Males coefficient of variation of force during elbow flexion across three testing days in the neutral and pronated forearm positions. The bar plots include data from all force levels (i.e. 2.5, 5, 10, 25, 50 and 75 %MVC).
